# A case in which all stones, including gallbladder stones as well as stacked bile duct stones, were removed using a transpapillary procedure

**DOI:** 10.1055/a-2706-3220

**Published:** 2025-10-21

**Authors:** Tomoya Takahashi, Hiroshi Ohyama, Yoshiki Ogane, Kazuki Watabe, Mayu Ouchi, Motoyasu Kan, Jun Kato

**Affiliations:** 1 Department of Gastroenterology, Graduate School of Medicine, University of Juntendo, Tokyo, Japan; 2 Department of Gastroenterology, Graduate School of Medicine, Chiba University, Chiba, Japan

The guidelines for the treatment of choledocholithiasis recommend that cholecystectomy should be performed after endoscopic removal of stones in the common bile duct (CBD) in cases with gallbladder stones 1 2 , but we report here on a case in which all stones in the CBD, intrahepatic bile duct, and gallbladder were removed through the papilla.

A 76-year-old man visited his previous doctor with nausea. He was diagnosed with acute cholangitis due to a large, stacked-type bile duct stone that had blocked the left and right intrahepatic bile ducts ( Fig. 1 ). However, the stone was difficult to remove, and he was referred to our hospital.

**Fig. 1:**
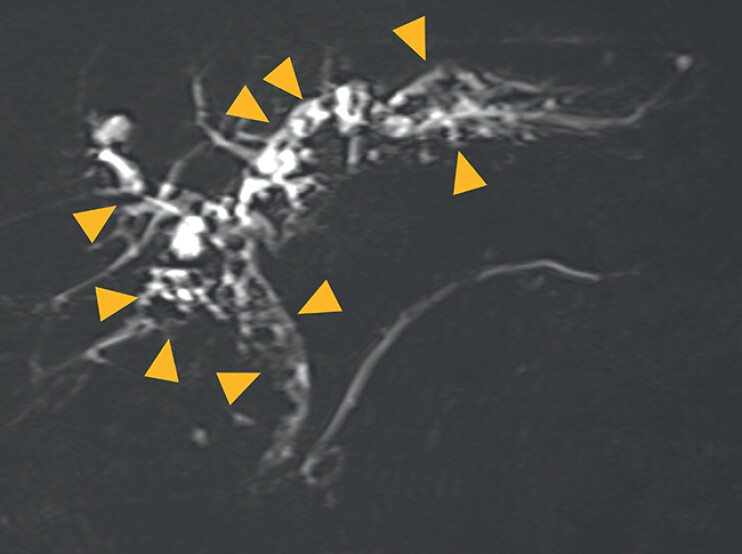
Magnetic resonance cholangiopancreatography showed stacked common bile duct stones (arrowheads).

First, we performed an endoscopic sphincterotomy combined with large-balloon dilation using a disposable balloon dilator. We then removed the distal bile duct stone using a stone removal balloon. Electrohydraulic lithotripsy was performed using a cholangioscope for the large stone at the cystic duct bifurcation. Then, we successfully removed giant stones at the confluence of the gallbladder ducts ( Fig. 2 ). As the cystic duct was large and a guidewire was successfully inserted, the stone was crushed using a stone-crushing basket, and a plastic stent was placed. In the re-treatment, endoscopic papillary large-balloon dilation was performed using a papilla dilation balloon, and the intrahepatic bile duct stone was removed using a balloon catheter. The stone at the bottom of the gallbladder remained, but the rotating basket (RASEN-2; Kaneka Medix, Osaka, Japan) was guided into the gallbladder, and the stone at the bottom of the gallbladder was removed ( Video 1 ). The cholangiogram confirmed that the stone had been completely removed.

**Fig. 2:**
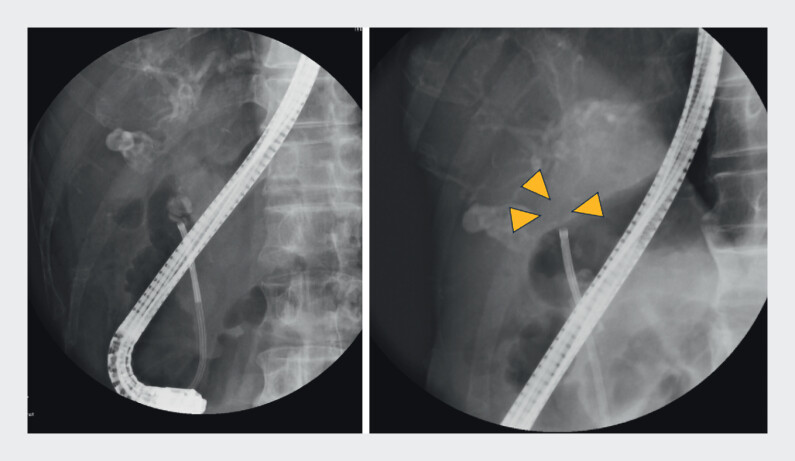
Electrohydraulic lithotripsy was performed using a cholangioscope for the large stone at the cystic duct bifurcation. We then successfully removed the giant stones at the confluence of the gallbladder ducts.

The stone at the bottom of the gallbladder remained, but the rotating basket was guided into the gallbladder, and the stone was removed.Video 1

Stones in the biliary tract can be removed entirely by transpapillary procedures. If the cystic duct is thick and straight, we might also attempt to remove the gallbladder stones.

Endoscopy_UCTN_Code_TTT_1AR_2AH

## Notes

Endoscopy E-Videos https://eref.thieme.de/e-videos
*E-Videos* is an open access online section of the journal *Endoscopy* , reporting on interesting cases and new techniques in gastroenterological endoscopy. All papers include a high-quality video and are published with a Creative Commons CC-BY license. Endoscopy E-Videos qualify for HINARI discounts and waivers and eligibility is automatically checked during the submission process. We grant 100% waivers to articles whose corresponding authors are based in Group A countries and 50% waivers to those who are based in Group B countries as classified by Research4Life (see: https://www.research4life.org/access/eligibility/ ). This section has its own submission website at https://mc.manuscriptcentral.com/e-videos .
